# Exploring Snapchat Dysmorphia, Body Dysmorphic Disorder Symptoms, and Body Trust in Patients Seeking Aesthetic Medicine Procedures

**DOI:** 10.1093/asj/sjaf185

**Published:** 2025-09-16

**Authors:** Paolo Mancin, Valentina Gaudenzi, Rossana Telesca, Domenico Centofanti, Emanuele Bartoletti, Silvia Cerea

## Abstract

**Background:**

Snapchat dysmorphia (SD) is an emerging phenomenon that characterizes individuals seeking aesthetic procedures to replicate the appearance of their digitally altered selfies. This phenomenon has been hypothesized to be linked to body dysmorphic disorder (BDD) symptoms. Additionally, body trust (ie, perceiving the body as safe and trustworthy, relying on its signals and sensations), which could contrast excessive focus on physical appearance, may moderate this relationship. Current literature on SD reveals a notable lack of comprehensive empirical investigations.

**Objectives:**

In this study, the authors examine the factorial structure and internal consistency of a newly developed measure: the SD Questionnaire (SDQ). Additionally, it explored the relationship between SD and BDD symptoms, with a focus on the potential moderating role of body trust.

**Methods:**

Data were collected from a sample of 163 women seeking aesthetic medicine treatments. The factorial structure and internal consistency of the SDQ were examined. Its association with BDD symptoms was explored within a hierarchical regression model, after controlling for other variables (eg, motivations to pursue cosmetic procedures). Finally, the potential moderating role of body trust was tested.

**Results:**

Findings supported a unidimensional factor structure for the SDQ, which also demonstrated a significant association with BDD symptoms. Body trust did not moderate this relationship.

**Conclusions:**

Findings provide preliminary support for the validity of the SDQ in women seeking aesthetic medicine procedures and lend empirical weight to anecdotal claims that SD is distinct yet related to BDD. Finally, although body trust was negatively associated with BDD symptoms, it did not show a moderation effect.

**Level of Evidence: 4 (Diagnostic):**

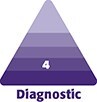

In the last decades, social media use has drastically increased.^1,2^ Among these platforms, scholars have identified a group that appears to exert a particularly negative influence on body image, which includes Instagram (Meta Platforms, Inc., Menlo Park, CA), Snapchat (Snap Inc., Santa Monica, CA), Pinterest (Pinterest, Inc., San Francisco, CA), Facebook (Meta Platforms, Inc.), and TikTok (TikTok Inc., Los Angeles, CA and Singapore).^3,4^ These social media are often referred to as appearance focused or image centric, because they primarily enable users to view and share photographs and videos that emphasize physical appearance.^5^

The widespread use of appearance-focused social media has contributed to the emergence of Snapchat dysmorphia (SD).^6^ First described in 2018 by plastic surgeon Tijion Esho, this phenomenon refers to individuals, particularly women, seeking cosmetic and aesthetic procedures from surgical and medical settings to resemble idealized versions of themselves, as observed in their own selfies digitally altered through filters, editing apps, and other photograph enhancement tools.^6-11^

These clients display unrealistic expectations because they believe that the “flawless” physical appearance portrayed in their modified selfie can be easily obtained through these procedures.^6^ SD can be described as a growing phenomenon. For instance, in the 2021 Annual American Academy of Facial Plastic and Reconstructive Surgery survey, 75% of surgeons have reported patients seeking cosmetic procedures to look better in selfies, which increased of 18% from 2018.^12,13^

SD appears as a complex phenomenon, characterized by an interplay of different factors, including involvement in appearance-focused social media use (eg, photograph manipulation/editing), beliefs about the possibility to achieve an idealized physical appearance, as the 1 obtained in the modified selfie, and susceptibility to other content portrayed online, such as content related to aesthetic or cosmetic interventions shared on social media.^6,8,9,14-16^

Especially after the COVID-19 pandemic, cosmetic and aesthetic medical professionals have reported a growing number of patients exhibiting characteristics of SD, raising concerns about the increasing prevalence and clinical relevance of this phenomenon.^7^  ^,8,14^ Despite this growing attention, SD has been discussed primarily in descriptive terms.^6,8,10,17^ Therefore, future research is needed to rigorously conceptualize and operationalize SD, including the development of comprehensive, empirically validated measures to assess it.

Scholars suggested that SD may be driven by extreme body dissatisfaction and, more specifically, that it is linked to body dysmorphic disorder (BDD) symptoms.^6,8,10,18^ BDD is a psychological disorder characterized by extreme body dissatisfaction, evidenced by excessive preoccupation with 1 or more, slightly visible or unobservable, perceived flaws in physical appearance, which causes clinically significant distress.^19^

A recent meta-analysis that included studies using structured diagnostic interviews reported a 20% prevalence of BDD among individuals in cosmetic and dermatology settings.^20^ Similar rates have been found among clients seeking minimally invasive procedures, such as botulinum toxin and injectable fillers. Specifically, the estimated prevalence of BDD risk is 15.8% to 24.7% in nonsurgical and aesthetic clinics, 12.5% in general dermatology, and 25% in cosmetic dermatology settings.^21-23^ Patients with BDD often seek cosmetic or aesthetic procedures to improve their physical appearance and reduce body dissatisfaction.^24,25^ However, these interventions frequently fail to resolve their underlying concerns.^26^ After undergoing treatment, many patients report exacerbation of BDD symptoms, dissatisfaction with the results, and the emergence of new appearance-related preoccupations.^27-30^ Nevertheless, some evidence suggests that patients with mild-to-moderate BDD symptoms, limited functional impairment, localized concerns, and realistic expectations may benefit from aesthetic procedures.^31,32^ These patients may report postoperative satisfaction and, in some cases, symptom remission.^33,34^

To date, evidence of an association between SD and BDD remains limited. Most available studies have investigated only specific aspects of this relationship, such as social media use and photograph manipulation, and have been conducted largely outside of aesthetic or cosmetic contexts.^35,36^ These findings suggest that social media use, especially photograph manipulation, may be a potential predictor of BDD.^35,36^ For instance, Wang et al found that the association between selfie editing and a positive attitude toward cosmetic surgery was mediated by facial dissatisfaction.^37^ However, because of the absence of comprehensive measures to assess SD, the nature of its relationship with BDD symptoms remains unclear. Therefore, more targeted research is needed to clarify this link, especially within cosmetic settings.

In exploring the relationship between SD and BDD, it is also essential to consider factors that might buffer or moderate the impact of SD on BDD symptoms. One such protective factor may be interoceptive awareness, particularly the component of body trust. Individuals with higher trust in their internal bodily sensations tend to report greater appreciation of their body functions, which in turn contributes to better well-being and a more positive body image.^38-41^ Trusting the body and its sensations may reduce reliance on external appearance-based cues, such as digitally modified selfies or idealized images shared on social media. This inward orientation (ie, focusing on how the body feels and functions) could weaken the association between SD and BDD symptoms.^42^

The authors of this study aim to address 2 primary objectives. The first objective was to develop and introduce a measure specifically designed to assess SD. For this purpose, we created the SD Questionnaire (SDQ), which was informed by existing descriptive evidence on this phenomenon.^6-8,10,14,17,43,44^ As part of the initial validation process, we explored its factorial structure and internal consistency.

The second objective was to investigate the relationship between BDD symptoms and SD, hypothesizing a positive association between these 2 constructs, as suggested by scholars describing SD.^8,10,18^ In addition, the authors of this study aim to assess the potential protective role of body trust in this relationship, an aspect that has not yet been explored in existing literature. Given that current research on SD is largely descriptive and lacks rigorous empirical investigation, to our knowledge, this is the first study to comprehensively examine the involvement of SD in BDD.

To achieve these objectives, a sample of women seeking aesthetic medicine interventions was recruited. This population was selected because of the higher prevalence of SD and elevated levels of body dissatisfaction and BDD symptoms observed among women.^8,20,29,45-50^ Women also tend to consider physical appearance a central component of their self-evaluation.^51-53^

## METHODS

The study was conducted in accordance with the Declaration of Helsinki and approved by the Ethics Committee of Psychological Research at the University of Padua. The study was conducted between March 2024 and February 2025. Participants (163 women seeking aesthetic medicine interventions) were recruited from aesthetic medicine clinics in Italy affiliated with the *Fondazione di Medicina Estetica Carlo Alberto Bartoletti*. Inclusion criteria to participate in the study were (1) to seek an aesthetic medicine procedure; (2) to engage in social media use and in photograph sharing; (3) to identify as women; and (4) to be at least 18 years old. Individuals interested in participating received an online link, developed utilizing Qualtrics (Qualtrics International Inc., Seattle [WA] and Provo [UT]), which was provided by an aesthetic medicine practitioner during consultations. The estimated time for the overall compilation was ∼10 min.

The link included the informed consent with information about the study's purposes, the voluntary nature of the participation, and the possibility to withdraw from the study without penalty. Participants were also asked to create a personal code with the first letters of their name and surname followed by their date of birth, for guaranteeing privacy and preventing duplicate compilations. Then, they completed a brief form (see Appendix A), designed to collect both sociodemographic information (ie, age, sex, gender, marital status, level of education, and occupational status) and information related to their current engagement with aesthetic medicine (ie, motivations for consulting an aesthetic medical professional, desired type of intervention, perceived flaws to correct, previous aesthetic medicine consultations, and past experience with aesthetic medicine and plastic surgery interventions). Following this information, participants were required to complete several self-report questionnaires: the SDQ, the Cosmetic Procedures Screening Questionnaire (COPS), the Beautification and the Emotional subscales of Cosmetic Motivations Questionnaire (CMQ), and the Trusting subscale of the Multidimensional Assessment of Interoceptive Awareness (MAIA).

The SDQ was developed based on the existing descriptive research on SD.^6-8,10,14,17,43,44^ This measure assesses both core features of SD—including involvement in photograph manipulation/editing, the desire to modify one's physical appearance to resemble that in selfies, and the influence of social media content and users on body image—as well as secondary aspects related to social media use in general, such as photograph sharing behaviors and the number of software/apps used for photograph manipulation/editing. Therefore, the SDQ provides both quantitative and qualitative information about SD in social media users.

The SDQ consists of 12 items divided into 3 blocks. The first block is made up of 7 items with different response formats. In the first item, raters chose from a list 1 or more social media they use to post their personal photographs, providing the number of social media in which photograph sharing takes place. Participants have the possibility to report “I do not post personal photos on social media” (SDQ 1). In the second item, participants are asked to describe the average time spent daily on social media, choosing from 7 options, ranging from “less than 10 minutes a day” to “6 hours or more a day”; participants have the possibility to report “I do not use social media” (SDQ 2). In the third item, they are required to select which software or apps they use to modify their self-photographs (more commonly referred to as “selfies”), providing the number of software and applications they used for photograph manipulation/editing. Participants have the option to select “I do not use software/apps to modify my photos” (SDQ 3). In the fourth item, they are asked to report the average number of selfies shared online in a week (SDQ 4). Closing the first block, 3 items with a Likert scale response format, ranging from 1 (“never”) to 10 (“always”), assess: (1) the frequency of photograph manipulation/editing before posting; (2) the degree of perceived influence of social media comments on their body image; and (3) the need to undergo aesthetic interventions experienced during photograph sharing. In the second block, which includes 4 items rated on a Likert scale from 1 (“not at all”) to 10 (“very much”), participants are asked to evaluate: (1) how feasible it would be to appear as in modified self-photographs; (2) whether they would like to look like their modified images; (3) whether time spent on social media influences their desire to undergo cosmetic procedures; and (4) whether the photographs and content they view impact this desire. The third block consists of a single item, also rated on a Likert scale from 1 (“definitely very low”) to 10 (“definitely very high”), in which participants self-report the extent to which they desire to look like their modified selfies. Although the first 4 items of the SDQ examined aspects associated with the phenomenon, the latter 8 items provide a score describing the presence of SD. The items of the SDQ are reported in Appendix B (Italian version) and Appendix C (English version).

The COPS is a screening measure that assesses the presence of BDD symptoms within cosmetic settings (eg, “How much do you feel your feature(s) is currently ugly, unattractive or ‘not right’?”; “How much does your feature(s) currently cause you a lot of distress?”).^54^ The COPS includes a preliminary question asking to describe the features that concern the raters, and the percentage of time spent worrying about it. The first area of concern that participants report should be the one that concerns them the most. Then, participants are asked to answer 9 items using a 9-point Likert scale, with values ranging from 0 to 8, whose continuum changes depending on the content of each item. The COPS can reach a maximum score of 72: high scores reflect greater impairment in functioning and a greater probability of BDD diagnosis.^54^ The COPS demonstrated a single factor and a good internal consistency (*α* = .91).^54^ The questionnaire also shows good test–retest reliability (*r* = 0.87; *P* < .01) and good convergent validity.^54^ Replicating Veale et al procedure, we conducted a Horn's parallel factor analysis to examine factor validity, confirming 1 factor for the Italian translation.^54^ Furthermore, the COPS demonstrated acceptable internal consistency (McDonald's *ω* = 0.84).

The Beautification and the Emotional subscales of the CMQ assess common motivations for undergoing cosmetic procedures.^55^ The Beautification subscale is composed of 7 items and was selected to assess the desire to improve one's physical attractiveness or keep up with current beauty trends (eg, “Look more like I do in filtered or edited images”).^55^ The Emotional subscale, composed of 5 items, assesses emotional or psychological aspects that contribute to the interest in cosmetic procedures (eg, “Feel more self-confident”); this subscale was selected to assess motivations to engage in cosmetic procedures typically associated with BDD.^55^ Each item is scored on a 3-point Likert scale, ranging from 0 (“does not apply to me”) to 2 (“applies to me very much”), and mean scores are calculated for each subscale, so that higher scores indicate greater identification with that motivational archetype.^55^ Internal consistency was acceptable for the Beautification subscale (McDonald's *ω* = 0.82), whereas it proved to be somewhat acceptable for the Emotional subscale (McDonald's *ω* = 0.65).

The 3-item Trusting subscale of the MAIA was employed to assess the experience of the body as safe and trustworthy (eg, “I feel my body is a safe place”; “I trust my body sensations”).^56,57^ This subscale is part of the MAIA, a self-report questionnaire that evaluates interoceptive awareness, comprising 32 items divided into 8 subscales, each of which assesses a specific dimension.^57^ Participants rate using a 6-point Likert scale, ranging from 0 (“never”) to 5 (“always”), how often each item refers to the person in everyday life. Mean scores are calculated for each subscale: higher scores indicate greater interoceptive awareness.^57^ The original English version of the MAIA shows appropriate internal consistency, with Cronbach's *α*s ranging from .66 to .87, and a good construct validity.^57^ The Italian version of the questionnaire replicated similar findings, such as Cronbach's *α*s that vary between .53 and .80 for each subscale.^56^ As for the Trusting subscale, it has demonstrated appropriate internal consistency in both the English (*α* = .79) and Italian versions (*α* = .80). Internal consistency of this subscale was found to be acceptable (McDonald's *ω* = 0.88) in the recruited sample.

### Statistical Analyses

Descriptive analyses were carried out. Means, standard deviations, and/or frequencies of the 4 items assessing features associated with SD were computed (Item 1 to Item 4). Then, we computed means, standard deviations, kurtosis, and skewness (cutoffs: skewness: ±2; kurtosis: ±7), and Pearson's correlations of the 8 items of the SDQ employed for the total score (Item 5 to Item 12).^58-60^ We also computed Mardia's test for multivariate normality: significant *P*-values for skewness and kurtosis would justify considering violation of normality in the choice of estimator for the Exploratory Factor Analysis (EFA) and the Confirmatory Factor Analysis (CFA). Then, we randomly split the data (*n* = 163) into 2 halves: the first half (*n* = 81) was employed to conduct an EFA, whereas the second half (*n* = 82) was employed to conduct a CFA.

Before conducting the EFA, we computed the Kaiser–Meyer–Olkin (KMO) and Bartlett's test of sphericity. The KMO should be at least ≥0.70 and Bartlett's test of sphericity should be significant to justify the application of an EFA.^61^ A visual analysis of the scree plot and eigenvalues >1 identified the appropriate number of factors to be fixed for the EFA.^62^ Principal Axis Factoring was chosen as an estimator for the EFA because it is more robust with violations of multivariate normality.^63^ Oblimin rotation was adopted. Items’ factor loadings were considered appropriate when >0.30. To evaluate the fit of the model, the model χ^2^, the Tucker–Lewis Index (TLI), and the Root Mean Square Error of Approximation (RMSEA) and its 90% CI were employed. For an adequate fit, the model χ^2^ should be nonsignificant, the TLI should show values ≥0.95, and the RMSEA should show values <0.08.^64^ The factorial structure identified with the EFA was tested with a CFA, conducted with maximum likelihood estimation with robust standard errors and a Satorra–Bentler (S-B) scaled test statistic. The same fit indices of the EFA were considered to assess the fit of the CFA. McDonald's omega was used to assess internal consistency: values should be >0.70.^65^ Finally, we explored Pearson's correlations between the SDQ scores and motivations to pursue cosmetic treatments (the subscales Emotional and Beautification of the CMQ). Correlations ≤0.10 were considered weak, ∼0.30 were considered moderate, and ∼0.50 were considered strong.^66^

To pursue the second objective, we computed Pearson's correlation to identify the variables to include in the regression models, considering age, BDD symptoms (ie, the COPS score), dimensions of the SD phenomenon (ie, the number of social media used for photograph sharing [SDQ 1], the number of software employed for photograph manipulation [SDQ 3], the number of weekly shared selfie [SDQ 4], and the SDQ total score), motivations to pursue cosmetic procedures (ie, the subscales Emotional and Beautification of the CMQ), and body trust (ie, the subscale Trusting of the MAIA). The variables that showed a significant correlation with the dependent variable (ie, the COPS score) were entered into hierarchical regression models. In the first step, we included aesthetic intervention-related variables, such as 2 dummy variables, identifying the desired type of cosmetic procedure (0 = “preventive”; 1 = “corrective”) and previous involvement in aesthetic procedures (0 = “no”; 1 = “yes”), and motivations to be involved in cosmetic procedures. Variables related to SD were considered in the second step. The Trusting subscale of the MAIA and the interaction term between the SDQ total score and this subscale were included, respectively, in the third and fourth steps. The regression models were supplemented with bootstrapped 95% CIs with 1000 replications.

## RESULTS

### Sample Characteristics

The sample consisted of 163 women seeking aesthetic medicine interventions (females: 100%), whose mean age was 43.66 (SD = 11.77; age range, 18-79 years). Concerning educational level, 1 participant completed primary school (0.6%), 4 lower secondary school (2.4%), 59 high school (36.2%), 94 bachelor and/or master's degree (57.7%), and 5 have followed alternative and/or postgraduate specialistic courses (3.1%). Pertaining to marital status, 31 individuals were single (19%), 20 had a fiancé or were in a nondomestic relationship (12.3%), 59 were married (36.2%), 34 were in a domestic relationship (20.9%), 4 were separated (2.4%), 12 were divorced (7.4%), and 3 were widowed (1.8%). As far as occupational status, 5 participants were students (3.1%), whereas 74.2% were full-time employed, employed, and/or self-employed, 9.8% part-time employed, 4.3% identified as housewives, 3.7% unemployed, 3.1% retired, and 1.8% fixed time/temporary employed.

Regarding the motivations to consult an aesthetic medical professional, 110 of them indicated “Feeling better about themselves” (67.5%), 49 “Feeling more beautiful” (30%), 41 “Physical maintenance” (25.1%), 32 “Rejuvenating” (19.6%), 30 “Increasing self-esteem” (18.4%), 15 “Improving professional and/or work image” (9.2%), 15 “Weight loss” (9.2%), 9 “Curiosity” (5.5%), 6 “Being more attractive to the partner” (3.7%), 5 “Post-pregnancy” (3.1%), and 2 “Post-menopause” (1.2%). Most of the sample (60.1%, *n* = 98) reported a desire for a corrective aesthetic intervention to address perceived blemishes, whereas the remaining participants (39.9%, *n* = 65) sought preventive treatments to avoid potential future physical flaws. When asked about the specific blemishes they wished to correct, 83 individuals (50.9%) answered facial wrinkles, 51 facial blemishes (31.3%), 47 adiposity (28.8%), 46 cellulite (28.2%), 40 cutaneous laxity (24.5%), 31 sunspots (19%), 25 obesity or overweight (15.3%), 10 venous-lymphatic insufficiency (6.1%), 7 capillaries (4.3%), 4 hypertrichosis (2.4%), 2 breasts (1.2%), and 1 hairline (0.6%). Overall, 118 participants (72.4%) had previously visited an aesthetic medical professional. Moreover, 104 participants (63.8%) reported having undergone at least 1 cosmetic procedure, with 102 having experienced an aesthetic medicine intervention and 33 having undergone plastic surgery.

### Factorial Structure and Internal Consistency of the Snapchat Dysmorphia Questionnaire (SDQ)

Mean, standard deviation, kurtosis and skewness, and correlations of the items of the SDQ employed for the total score are displayed in Table 1. Collectively, the items were considered appropriate for factorial analyses. Notably, Items 10 and 11 demonstrated a strong correlation (*r* = 0.93). After controlling the content of these items, we agreed that participants should have interpreted them similarly; therefore, we decided to remove Item 11 from the analysis, reducing redundancy (Table 1). Mardia's test for multivariate normality was significant for both skewness and kurtosis (all *P* < .001), highlighting a violation of multivariate normality.

**Table 1.** Means, Standard Deviations, Skewness, Kurtosis, and Correlations of Each Item Employed for the Total Score of the Snapchat Dysmorphia Questionnaire

	Mean (SD)	Skewness	Kurtosis	1	2	3	4	5	6	7
1. SDQ 5	2.30 (2.46)	1.92	2.61							
2. SDQ 6	3.07 (2.77)	1.16	0.12	0.37***						
3. SDQ 7	3.56 (3.04)	0.87	−0.63	0.27***	0.54***					
4. SDQ 8	2.60 (2.30)	1.42	1.14	0.42***	0.41***	0.37***				
5. SDQ 9	4.13 (3.24)	0.59	−1.09	0.59***	0.47***	0.52***	0.49***			
6. SDQ 10	3.23 (2.92)	1.04	−0.32	0.43***	0.44***	0.57***	0.40***	0.57***		
7. SDQ 11	3.54 (2.90)	0.86	−0.56	0.41***	0.41***	0.62***	0.37***	0.59***	0.93***	
8. SDQ 12	3.35 (2.81)	0.96	−0.36	0.54***	0.54***	0.58***	0.50***	0.74***	0.64***	0.66***

SDQ, Snapchat Dysmorphia Questionnaire;

^***^ *P* < .001.

**Figure 1.**
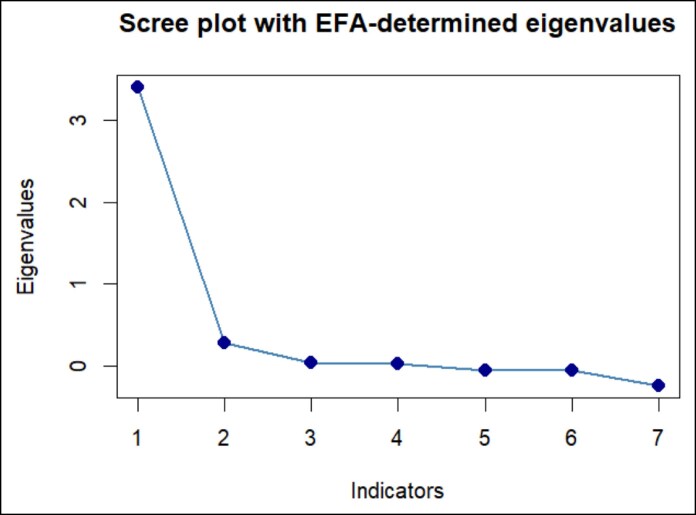
Scree plot of the Snapchat Dysmorphia Questionnaire.

The first half of the sample was used to conduct an EFA: KMO was 0.86 and Bartlett's test of sphericity was significant (*P* < .001), justifying the application of an EFA. Both the analysis of the eigenvalues and the scree plot (Figure 1) suggested that a single factor would be appropriate.

The EFA with 1 fixed factor resulted in a satisfactory fit: model χ^2^(14) = 20.45; *P* = .12; RMSEA = 0.07 (90% CI, <0.001, 0.14); TLI = 0.95. All factor loadings were >0.30 (Table 2); the single factor explained 49% of variance in the measure. Hence, we conducted a CFA adopting Maximum Likelihood Estimation with robust standard errors and an S-B scaled test statistic fixing a single factor. Overall, the model had a good fit, supporting a single factor for the SDQ: scaled χ^2^(14) = 13.96, *P* = .45; S-B correction: 1.75; TLI = 1.00; RMSEA < 0.001 (90% CI, <0.001, 0.14). Factor loadings are presented in Table 2. The McDonald's omega of the SDQ was 0.90 (Table 2).

**Table 2.** Factor Loadings of EFA and the CFA of Each Item of the Snapchat Dysmorphia Questionnaire Employed for the Total Score

Items	EFA	CFA
SDQ 5	0.58	0.64
SDQ 6	0.66	0.61
SDQ 7	0.67	0.70
SDQ 8	0.68	0.52
SDQ 9	0.76	0.87
SDQ 10	0.67	0.78
SDQ 12	0.83	0.92

CFA, Confirmatory Factor Analysis; EFA, Exploratory Factor Analysis; SDQ, Snapchat Dysmorphia Questionnaire.

The SDQ total score showed positive correlations with the number of social media used for photograph sharing (*r* = 0.24, *P* = .002), the number of software employed for photograph manipulation (SDQ 3) (*r* = 0.55, *P* < .001), and the number of weekly shared selfie (SDQ 4) (*r* = 0.31, *P* < .001). Frequencies of Item 2 of the SDQ are reported in Appendix D.

### Associations Between Body Dysmorphic Disorder (BDD) Symptoms and SD and Possible Moderation of Body Trust

According to correlations (Table 3), we included 2 dummy variables (the desired type of procedure—corrective or preventive—and previous involvement in aesthetic medicine and plastic surgery interventions) and the subscale Emotional and Beautification of the CMQ in the first step of the model. The first step was statistically significant (*P* < .001): the desired type of procedure (corrective) and both motivations to pursue cosmetic treatments emerged as possible predictors of BDD symptoms, explaining 44% of variance. In the second step, we included SD and its associated features (SDQ 3, SDQ 4, and the SDQ total score). The second step emerged as statistically significant (*P* < .001): the desired type of procedure (corrective), the motivation to pursue cosmetic treatments to cope with emotional or psychological distress, and SD emerged as possible predictors of BDD symptoms. The overall model explained 47% of variance, including the variables related to SD explain an additional 3% of variance in the model (*F*(3) = 2.88; *P* = .03). In the third step, we included body trust (the subscale Trusting of the MAIA). This model was statistically significant (*P* < .001): the motivations to pursue cosmetic treatments and SD emerged as positively associated with BDD symptoms, whereas body trust emerged as negatively associated. Collectively, these variables explained 54% of variance: the subscale Trusting of the MAIA explained an additional 7% of variance in the model (*F*(1) = 22.91; *P* < .001). In the fourth step, the interaction term between the SDQ total score and the subscale Trusting of the MAIA emerged as statistically significant; however, after considering bootstrapped CI, this variable proved to be nonsignificant. Therefore, moderation was not supported. The results of the hierarchical regression model are presented in Table 4.

**Table 3.** Means, Standard Deviations, and Correlations of Age, SDQ Scores, the COPS Total Score, CMQ Scores, and the Subscale MAIA Subscale

	Age	SDQ 1	SDQ 3	SDQ 4	SDQ total score	CMQ: beautification scale	CMQ: emotional scale	MAIA: trusting scale	Mean (SD)
COPS total score	−0.15	0.09	0.21**	0.17*	0.53***	0.48***	0.61***	−0.50***	17.58 (11.20)
Mean (SD)	43.66 (11.77)	1.91 (1.12)	0.80 (1.05)	1.37 (2.42)	3.18 (2.12)	0.73 (0.44)	0.99 (0.46)	3.36 (1.21)	

COPS, Cosmetic Procedures Screening Questionnaire; CMQ, Cosmetic Motivations Questionnaire; MAIA, Multidimensional Assessment of Interoceptive Awareness; SDQ, Snapchat Dysmorphia Questionnaire.

^*^ *P* < .05,

^**^ *P* < .01,

^***^ *P* < .001.

**Table 4.** Results of Hierarchical Regression Analysis Including Body Dysmorphic Disorder Symptoms as a Dependent Variable and the Moderation of Body Trust

						Bootstrapped 95% CI			
Variables	B	SE	*β*	*t*	*P*	LL	UL	*F*	df
Step 1								30.58***	4158
Intercept	1.06	2.00	—	0.53	.60	−2.88	5.44		
Type of desired intervention	3.10	1.36	.17	2.87	.005	0.97	6.39		
Previous involvement in cosmetic procedures	−2.20	1.40	−.09	−1.58	.11	−5.01	0.44		
CMQ: Beautification scale	5.27	1.87	.21	2.82	.005	1.98	8.98		
CMQ: Emotional scale	11.88	1.80	.48	6.59	<.001	8.16	15.90		
Step 2								19.33***	7155
Intercept	0.91	1.97	—	0.46	.64	−2.67	5.03		
Type of desired intervention	3.18	1.38	.14	2.30	.02	0.50	5.74		
Previous involvement in cosmetic procedures	−2.00	1.38	−.09	−1.45	.15	−4.71	0.61		
CMQ: Beautification scale	2.93	2.04	.12	1.44	.15	−1.14	6.98		
CMQ: Emotional scale	10.56	1.85	.43	5.70	<.001	6.29	14.15		
SDQ 3	−0.64	0.81	−.06	−0.79	.43	−2.77	1.03		
SDQ 4	−0.18	0.31	−.04	−0.59	.55	−1.09	0.88		
SDQ total score	1.33	0.47	.25	2.83	.005	0.36	2.53		
Step 3								22.17***	8154
Intercept	12.58	3.06	—	4.12	<.001	5.60	19.42		
Type of desired intervention	2.03	1.32	.09	1.55	.12	−0.50	4.75		
Previous involvement in cosmetic procedures	−1.25	1.30	−.05	−0.96	.34	−3.94	1.51		
CMQ: Beautification scale	3.89	1.92	.15	2.03	.04	0.17	7.54		
CMQ: Emotional scale	7.85	1.82	.32	4.31	<.001	3.52	12.06		
SDQ 3	−0.64	0.75	−.06	−0.85	.39	−2.24	0.94		
SDQ 4	−0.11	0.29	−.02	−0.37	.71	−0.90	0.77		
SDQ total score	1.17	0.44	.22	2.64	.009	0.23	2.24		
MAIA: Trusting scale	−2.70	0.56	−.29	−4.79	<.001	−3.91	−1.34		
Step 4								20.68***	9153
Intercept	7.11	3.95	—	1.80	.07	−1.56	16.12		
Type of desired intervention	1.87	1.30	.08	1.44	.15	−0.76	4.46		
Previous involvement in cosmetic procedures	−1.03	1.29	−.04	−0.80	.43	−3.55	1.68		
CMQ: Beautification scale	4.12	1.90	.16	2.17	.03	0.22	7.69		
CMQ: Emotional scale	7.77	1.80	.32	4.31	<.001	3.32	11.60		
SDQ 3	−0.49	0.75	−.05	−0.65	.52	−2.21	1.11		
SDQ 4	−0.12	0.29	−.03	−0.42	.67	−0.95	0.68		
SDQ total score	2.77	0.86	.52	3.21	.002	0.40	4.97		
MAIA: Trusting scale	−1.10	0.93	−.12	−1.18	.24	−3.21	1.00		
MAIA: Trusting scale × SDQ total score	−0.52	0.24	−.34	−2.15	.03	−1.12	0.18		

CMQ, Cosmetic Motivations Questionnaire; COPS, Cosmetic Procedures Screening Questionnaire; MAIA, Multidimensional Assessment of Interoceptive Awareness; SDQ, Snapchat Dysmorphia Questionnaire.

^***^ *P* < .001.

## DISCUSSION

In this study, the authors provide preliminary support for the use of the SDQ in assessing SD in a sample of women seeking aesthetic medicine procedures. Findings provided by the EFA supported 1 factor structure, which was replicated by conducting the CFA: all items loaded coherently onto a single factor. Thus, the SDQ captures various features of SD as a single construct, comprising engagement in photograph manipulation/editing and its influence on the desire to modify physical appearance, beliefs about the attainability of the appearance portrayed in altered images, and the influence of the social media environment on the desire to be involved in aesthetic interventions.^6-8,10,14,17,43,44^ Reliance on a single factor is also supported by the excellent internal consistency (McDonald's *ω* = 0.90). Further supporting the potential utility of this measure, the total SDQ score demonstrated moderate-to-strong correlations with other indices provided by the SDQ, such as the number of social media used for photograph sharing, the average number of selfies weekly shared, and the number of software/apps used for photograph manipulation/editing. Collectively, these findings supported SD as a unitary phenomenon, as suggested by other scholars.^6,10,14^

As for the second objective, the third step of the hierarchical regression model highlighted that BDD symptoms were positively associated with both examined motivations to pursue cosmetic procedures and SD, while being negatively associated with body trust. Specifically, BDD symptoms were found to be significantly associated with motivations for pursuing cosmetic treatments aimed at improving attractiveness and coping with emotional and psychological distress. This finding aligns with existing evidence showing a high prevalence of clients with BDD symptoms in aesthetic settings:^20,23,67^ women with these symptoms often seek aesthetic enhancements as a solution to their preoccupation with physical appearance, believing that these interventions will address perceived flaws and improve their appearance.^24,25^ The observed association between SD and BDD symptoms, after controlling for other aesthetic-intervention-related variables, suggests a potential influence of SD on BDD symptoms among women seeking aesthetic treatments, as initially hypothesized.^8,10,14^ Therefore, SD could potentially serve as a risk factor for BDD. Moreover, it provided evidence of this phenomenon among women attending aesthetic settings: SD may play a role in shaping their decision-making process regarding aesthetic enhancements.^6^ However, it is important to note that the increase in explained variance after including SDQ scores in the model was relatively small (Δ*R*^2^ = 0.03), suggesting that the influence of SD on BDD symptoms may be marginal. This indicates that although a significant association between SD and BDD symptoms was observed, SD might represent only one of the multiple contributing factors in the complex etiology of BDD symptoms. Further research is therefore needed to better understand the extent and mechanisms of this relationship.

Although the influence of SD on BDD symptoms appeared to be marginal, the findings of the study pointed to the relevance of a different, protective, psychological dimension—body trust—which was negatively associated with BDD symptoms. This is in line with previous literature suggesting that BDD symptoms are characterized by the processing of external signals rather than internal ones: there is an excessive focus on how the body looks rather than how it feels or functions.^68-70^ This process is also especially prominent among women because of pervasive sociocultural pressures that foster self-objectification.^71^ Being able to trust the body and its cues may favor an internal body orientation and distance from these negative processes, ultimately serving as a possible protective factor for BDD symptoms.^72^ Despite the promising findings, results from the fourth regression model showed that body trust did not moderate the relationship between BDD symptoms and SD. One possible explanation lies in the specific characteristics of the recruited sample—women actively seeking aesthetic medicine procedures—who may already be highly oriented toward physical appearance. In such a population, body trust alone might not suffice to counteract the strong sociocultural influences shaping their body image. This highlights the complexity of protective mechanisms in the context of negative body image and calls for further exploration into appearance-focused populations, such as women seeking cosmetic procedures.

### Limitations and Future Directions

These findings should be considered in light of several limitations. To the best of our knowledge, this is the first study attempting to develop a measure to assess SD. Therefore, it was not possible to include similar measures to evaluate convergent validity. Moreover, the authors of this study provided support for the use of the SDQ relying only on a sample of women seeking aesthetic medicine procedures, limiting the generalizability of these findings to other settings and populations (eg, men). Neither the COPS nor the CMQ is validated for the Italian population: these measures were selected because of their appropriateness for the study and their broad use in research. Moreover, the subscale Emotional of the CMQ demonstrated somewhat low internal consistency, which could potentially undermine the results. The study is cross-sectional; therefore, cause-and-effect relationships may be only suggested. Lack of a control group, composed of women recruited from the general population, could clarify whether these findings are specific for the intended population.

Future studies should assess other psychometric properties of the SDQ, such as temporal stability, to provide further support on the validity of this measure. Moreover, increasing the sample size and including more diversity in terms of age, gender, and cultural background would corroborate and broaden these results. A longitudinal design, following a sample of women before and after aesthetic treatments, would enable to clarify cause-and-effects relationships and to evaluate the influence of SD on postintervention satisfaction. Because body trust did not emerge as a moderator in the relationship between BDD symptoms and SD, other potential protective variables could be considered for this role. For example, components of positive body image, such as body and functionality appreciation, could be viable candidates.^73,74^

## CONCLUSIONS

To the best of our knowledge, this is the first study to provide a measure assessing SD. Moreover, it is the first study to investigate the relationship between this phenomenon and BDD symptoms, considering the potential protective role of body trust. According to our findings, the SDQ provides a valid score to assess the SD phenomenon among women seeking aesthetic medicine interventions. Moreover, SD emerged as a complex phenomenon, and is associated with BDD symptoms. Finally, body trust was negatively associated with BDD symptoms, but it did not moderate the expected association.

## Supplemental Material

This article contains supplemental material located online at https://doi.org/10.1093/asj/sjaf185.

## Supplementary Material

sjaf185_Supplementary_Data
