# Supplementary material for: Exploring Snapchat Dysmorphia, Body Dysmorphic Disorder Symptoms, and Body Trust in Patients Seeking Aesthetic Medicine Procedures
Source: Aesthet Surg J. 2025 Sep 16;46(2):213–21. doi: 10.1093/asj/sjaf185 (PMC12853383; doi:10.1093/asj/sjaf185)
Supplement: sjaf185_Supplementary_Data [file sjaf185_supplementary_data.zip › Appendix A.docx]

**Appendix A.** Socio-demographic information.

Personal code (please, create a personal code using the first letters of your name and surname followed by your date of birth):

Gender:

- Woman
- Man
- Other (specify)

Sex at birth:

- Female
- Male
- Other (specify)

Age:

Educational level:

- Primary school
- Secondary school
- High school
- Bachelor and/or Master’s degree
- Other (specify)

Marital status:

- Single
- Fiancé and/or non-domestic relationship
- Married
- Domestic relationship
- Separated
- Divorced
- Widowed

Occupational status:

- Student
- Full-time employed, employed and/or self-employed
- Part-time employed
- Housewife
- Unemployed
- Retired
- Fixed time/temporary employed
- Other (specify)

**Aesthetic medicine-related information**

Why are you consulting an aesthetic medicine practitioner?

- Feeling better about myself
- Being more attractive to the partner
- Curiosity
- Rejuvenating
- Feeling more beautiful
- Improving professional and/or work image
- Physical maintenance
- Weight loss
- Increasing self-esteem
- Post-pregnancy
- Post-menopause
- Other (specify)

What type of aesthetic intervention do you desire to pursue:

- Preventive (to avoid potential future physical flaws)
- Corrective (to address perceived blemishes)

What type of blemishes do you wish to correct to improve your appearance?

- Localized adiposity
- Cellulite
- Obesity or overweight
- Facial wrinkles
- Cutaneous laxity
- Hypertrichosis
- Sunspots
- Venous-lymphatic insufficiency
- Capillaries
- Facial blemishes
- Baldness
- Other (specify)

Have you previously consulted an aesthetic medical professional?

- Yes
- No

Have you ever undergone an aesthetic medical intervention?

- Yes
- No

Have you ever undergone a plastic surgery intervention?

- Yes
- No
