# Supplementary material for: Exploring Snapchat Dysmorphia, Body Dysmorphic Disorder Symptoms, and Body Trust in Patients Seeking Aesthetic Medicine Procedures
Source: Aesthet Surg J. 2025 Sep 16;46(2):213–21. doi: 10.1093/asj/sjaf185 (PMC12853383; doi:10.1093/asj/sjaf185)
Supplement: sjaf185_Supplementary_Data [file sjaf185_supplementary_data.zip › Appendix B.docx]

Appendix B. SDQ

Le seguenti domande fanno riferimento alle sue fotografie condivise/pubblicate sui *social media*. Le chiediamo di rispondere facendo riferimento ai ***selfie*** e alle **fotografie che la ritraggono**, ovvero in cui viene fotografato/a per intero o in parte, e che vengono pubblicate sui *social media*.

# I BLOCCO

1. Indichi su quali *social media* pubblica/condivide le sue fotografie selezionando una o più delle seguenti opzioni:
   - Instagram
   - Facebook
   - Snapchat
   - TikTok
   - BeReal
   - Whatsapp
   - Pinterest
   - LinkedIn
   - Altro (specificare)
   - Non pubblico fotografie personali sui *social media*
2. Quanto tempo in media ha trascorso, **giornalmente**, **nel corso dell’ultimo mese**, sui *social media*? (Solo una risposta ammessa)

O Meno di 10 minuti al giorno

O 10-30 minuti al giorno O 30-60 minuti al giorno O 1-2 ore al giorno

O 2-3 ore al giorno

O 3-5 ore al giorno

O 6 ore o più al giorno

O Non utilizzo i *social media*

1. Indichi quali *software* o applicazioni utilizza per modificare le sue fotografie selezionando una o più delle seguenti opzioni:
   - Filtri fotografici già disponibili sul mio cellulare/*tablet*
   - Filtri disponibili sul/sui *social media* in cui condivido le mie fotografie
   - CapCut – Editor video e foto
   - Remini - Migliora Foto con AI
   - PicsArt AI Modifica Foto
   - FaceApp: Editor viso perfetto
   - Photoshop Express Fotomontaggi
   - Filtri foto Photoshop Camera
   - Adobe Photoshop Lightroom foto ritocco
   - VSCO: Editor di Foto e Video
   - Facetune: Modifica Foto e IA
   - InStories Reels & Story Maker
   - YouCam Perfet: Photo Editor
   - YouCam Makeup: Beauty Selfie
   - PhotoDirector – Editor di Foto
   - Peachy – Face & Body Editor
   - BeautyPlus – AI Photo Editor
   - Airbrush – Editor di foto AI
   - Altro (specificare)
   - Non utilizzo *software*/applicazioni per modificare le mie fotografie
2. Quante fotografie in media ha pubblicato/condiviso **in una settimana, nel corso dell’ultimo mese**, sui *social media*? Indichi il numero (anche se non è preciso): alla settimana
3. Nel complesso, quanto frequentemente modifica le sue fotografie (ad esempio, con i filtri) prima di pubblicarle/condividerle sui *social media*?

| 1 | 2 | 3 | 4 | 5 | 6 | 7 | 8 | 9 | 10 |
| --- | --- | --- | --- | --- | --- | --- | --- | --- | --- |
| Mai |  |  |  |  |  |  |  |  | Sempre |

1. Quanto si sente influenzata/o dai commenti sul suo aspetto fisico che riceve sui *social media*?

| 1 | 2 | 3 | 4 | 5 | 6 | 7 | 8 | 9 | 10 |
| --- | --- | --- | --- | --- | --- | --- | --- | --- | --- |
| Mai |  |  |  |  |  |  |  |  | Sempre |

1. Ha mai sentito il bisogno di svolgere interventi estetici per modificare il suo aspetto fisico guardando le fotografie che sta per condividere/che ha appena condiviso sui *social media*?

| 1 | 2 | 3 | 4 | 5 | 6 | 7 | 8 | 9 | 10 |
| --- | --- | --- | --- | --- | --- | --- | --- | --- | --- |
| Mai |  |  |  |  |  |  |  |  | Sempre |

# II BLOCCO

1. Pensa che sia possibile apparire nella realtà come nelle fotografie modificate (ad esempio, attraverso i filtri) e pubblicate/condivise sui *social media*?

1 2 3 4 5 6 7 8 9 10

Per nulla

Moltissimo

1. Le piacerebbe che il suo aspetto fisico fosse simile a quello delle sue fotografie modificate (ad esempio, attraverso i filtri) e che pubblica/condivide sui *social media*?

1 2 3 4 5 6 7 8 9 10

Per nulla

Moltissimo

1. Passare il tempo sui *social media* influenza il suo desiderio di sottoporsi a interventi estetici per modificare il suo aspetto fisico?

1 2 3 4 5 6 7 8 9 10

Per nulla

Moltissimo

1. Le fotografie e i contenuti che guarda sui *social media* influenzano il suo desiderio di sottoporsi a interventi estetici per modificare il suo aspetto fisico?

1 2 3 4 5 6 7 8 9 10

Per nulla

Moltissimo

# III BLOCCO

1. Quanto è forte il suo desiderio di apparire come nelle sue fotografie modificate con i filtri?

1 2 3 4 5 6 7 8 9 10

Decisamente molto basso

Decisamente molto alto
