# Supplementary material for: Exploring Snapchat Dysmorphia, Body Dysmorphic Disorder Symptoms, and Body Trust in Patients Seeking Aesthetic Medicine Procedures
Source: Aesthet Surg J. 2025 Sep 16;46(2):213–21. doi: 10.1093/asj/sjaf185 (PMC12853383; doi:10.1093/asj/sjaf185)
Supplement: sjaf185_Supplementary_Data [file sjaf185_supplementary_data.zip › Appendix C.docx]

Appendix C. SDQ

The following questions refer to your photos, shared/posted on social media. Please, while answering, refer to **selfies** and to **photos that portray you**, in which you are depicted either fully or partially, and that are posted on social media.

# I BLOCK

1. Indicate on which social media you post/share your photos by choosing one or more of the following options:
   - Instagram
   - Facebook
   - Snapchat
   - TikTok
   - BeReal
   - Whatsapp
   - Pinterest
   - LinkedIn
   - Other (specify)
   - I do not post personal photos on social media
2. On average, how much time **per day** have you spent on social media **over the past month**? (Only one answer allowed)

O Less than 10 minutes a day

O 10-30 minutes a day O 30-60 minutes a day O 1-2 hours a day

O 2-3 hours a day

O 3-5 hours a day

O 6 hours or more a day

O I do not use social media

1. Indicate which software or app you use to modify your photos by choosing one or more of the following options:
   - Filters already available on my phone/tablet
   - Filters available on the social media I use to share my photos
   - CapCut - Video Editor
   - Remini – AI Photo Enhancer
   - Picsart AI Photo Editor, Video
   - FaceApp: Perfect Face Editor
   - Photoshop Express Photo Editor
   - Photoshop Camera
   - Lightroom Photo & Video Editor
   - VSCO: Photo Editor
   - Facetune: Video & Photo Editor
   - InStories Reels & Story Maker
   - YouCam Perfect: Photo Editor
   - YouCam Makeup: Face Editor
   - PhotoDirector: AI Photo Editor
   - Peachy – Face & Body Editor
   - BeautyPlus – AI Photo Editor
   - Airbrush: Face & Tan Editor
   - Other (specify)
   - I do not use software/apps to modify my photos
2. On average, how many photos **per week** have you posted/shared on social media **over the past month**? Report the number (even if it is not precise): a week
3. Overall, how frequently do you modify your photos (for example, with filters) before posting/sharing them on social media?

| 1 | 2 | 3 | 4 | 5 | 6 | 7 | 8 | 9 | 10 |
| --- | --- | --- | --- | --- | --- | --- | --- | --- | --- |
| Never |  |  |  |  |  |  |  |  | Always |

1. How much do you feel influenced by comments on your appearance received on social media?

| 1 | 2 | 3 | 4 | 5 | 6 | 7 | 8 | 9 | 10 |
| --- | --- | --- | --- | --- | --- | --- | --- | --- | --- |
| Never |  |  |  |  |  |  |  |  | Always |

1. Have you ever felt the need to undergo aesthetic interventions to modify your appearance looking at the photos you are about to share/you have just shared on social media?

| 1 | 2 | 3 | 4 | 5 | 6 | 7 | 8 | 9 | 10 |
| --- | --- | --- | --- | --- | --- | --- | --- | --- | --- |
| Never |  |  |  |  |  |  |  |  | Always |

# II BLOCK

1. Do you think it is possible to appear in real life as in the modified photos (for example, with filters), posted/shared on social media?

1 2 3 4 5 6 7 8 9 10

Not at all

Very much

1. Would you like your appearance to look more like it does in the modified photos (e.g., with filters) that you post/share on social media?

1 2 3 4 5 6 7 8 9 10

Not at all

Very much

1. Does spending time on social media influence your desire to undergo aesthetic procedures to modify your appearance?

1 2 3 4 5 6 7 8 9 10

Not at all

Very much

1. Do the photos and content that you see on social media influence your desire to undergo aesthetic procedures to modify your appearance?

1 2 3 4 5 6 7 8 9 10

Not at all

Very much

# III BLOCK

1. How strong is your desire to appear as in your photos that have been modified with filters?

1 2 3 4 5 6 7 8 9 10

Definitely very low Definitely very high
