# Supplementary material for: Exploring Snapchat Dysmorphia, Body Dysmorphic Disorder Symptoms, and Body Trust in Patients Seeking Aesthetic Medicine Procedures
Source: Aesthet Surg J. 2025 Sep 16;46(2):213–21. doi: 10.1093/asj/sjaf185 (PMC12853383; doi:10.1093/asj/sjaf185)
Supplement: sjaf185_Supplementary_Data [file sjaf185_supplementary_data.zip › Appendix D.docx]

**Appendix D.** Frequencies of the item 2 of the SDQ.

| Options | Frequency |
| --- | --- |
| Less than 10 minutes a day | 23 |
| 10-30 minutes a day | 45 |
| 30-60 minutes a day | 38 |
| 1-2 hours a day | 32 |
| 2-3 hours a day | 16 |
| 3-5 hours a day | 4 |
| 6 or more hours a day | 5 |
